# Rethinking Rice Preparation for Highly Efficient Removal of Inorganic Arsenic Using Percolating Cooking Water

**DOI:** 10.1371/journal.pone.0131608

**Published:** 2015-07-22

**Authors:** Manus Carey, Xiao Jiujin, Júlia Gomes Farias, Andrew A. Meharg

**Affiliations:** 1 Institute for Global Food Security, Queen’s University Belfast, Belfast, Northern Ireland; 2 Sichuan Agricultural University, Chengdu, Sichuan, P. R. China; 3 Departamento de Biologia, Centro de Ciências Naturais e Exatas, Universidade Federal de Santa Maria, Santa Maria, Brazil; University of Louisville, UNITED STATES

## Abstract

A novel way of cooking rice to maximize the removal of the carcinogen inorganic arsenic (As_*i*_) is presented here. In conventional rice cooking water and grain are in continuous contact, and it is known that the larger the water:rice cooking ratio, the more As_*i*_ removed by cooking, suggesting that the As_*i*_ in the grain is mobile in water. Experiments were designed where rice is cooked in a continual stream of percolating near boiling water, either low in As_*i*_, or As_*i*_ free. This has the advantage of not only exposing grain to large volumes of cooking water, but also physically removes any As_*i*_ leached from the grain into the water receiving vessel. The relationship between cooking water volume and As_*i*_ removal in conventional rice cooking was demonstrated for the rice types under study. At a water-to-rice cooking ratio of 12:1, 57±5% of As_*i*_ could be removed, average of 6 wholegrain and 6 polished rice samples. Two types of percolating technology were tested, one where the cooking water was recycled through condensing boiling water steam and passing the freshly distilled hot water through the grain in a laboratory setting, and one where tap water was used to cook the rice held in an off-the-shelf coffee percolator in a domestic setting. Both approaches proved highly effective in removing As_*i*_ from the cooking rice, with up to 85% of As_*i*_ removed from individual rice types. For the recycled water experiment 59±8% and 69±10% of As_*i*_ was removed, on average, compared to uncooked rice for polished (n=27) and wholegrain (n=13) rice, respectively. For coffee percolation there was no difference between wholegrain and polished rice, and the effectiveness of As_*i*_ removal was 49±7% across 6 wholegrain and 6 polished rice samples. The manuscript explores the potential applications and further optimization of this percolating cooking water, high As_*i*_ removal, discovery.

## Introduction

Paddy rice is the carbohydrate staple of half the world yet is the main source of exposure to the class-one, non-threshold carcinogen inorganic arsenic (As_*i*_) [1]. International and national bodies are in the process of setting standards for inorganic arsenic in rice due to the fact that sub-populations are exposed to levels that are associated with negative health consequences [2]. The UN WHO has just set, in 2014, advisory levels of As_*i*_ in polished (*i*.*e*. white) rice grain at 0.2 mg/kg [3], while the European Union [2] and United States of America [4] are in the process of setting legal standards for inorganic arsenic in rice based products.

It is highly desirable to reduce the inorganic arsenic content of rice. While breeding low arsenic rice and altering of rice cultivation practice offer medium to long-term solutions to reducing inorganic arsenic content of grain [5], if rice preparation to remove inorganic arsenic is optimized, this offers an immediate solution to decreasing inorganic arsenic in the diet. Milling, fortuitously for those who prefer polished (white rice), already reduces the inorganic arsenic content of rice by ~50% [6,7]. Bran is much more enriched (~10-fold) in As_*i*_ compared to polished (white) rice [6,7], though the milled bran component is only ~10% of wholegrain biomass. However, the levels of As_*i*_ in polished rice are still highly problematic [1,4,6,7]. The way that rice is cooked has been shown to have an effect on As_*i*_ content. The now common way to cook boiled rice throughout the globe is to use a volume of water that will result in all the water being absorbed or evaporated. This does not cause loss of As_*i*_ on cooking [8–10]. Where the cooking water itself is elevated in As_*i*_, such as in large tracts of Bangladesh, cooking further elevates the arsenic content of the cooked product [11]. Traditional S.E. Asian methods of rice cooking involved extensive rinsing of the uncooked grain followed by cooking the rice in a large excess of water and discarding that water on cessation of cooking and this was found to reduce As_*i*_ content of food by up to 45% [10] and 57% [8] when As_*i*_ free water was used. Steaming is another traditional cooking approach, where rice is not directly exposed to cooking water, but steaming can only remove up ~10% of As_*i*_ [10].

In the study presented here rice cooking is radically rethought to optimize As_*i*_ removal. It is demonstrated that percolating technology, where cooking water, either free off, or low in, As_*i*_ is continually passed through rice in a constant flow, is highly efficient at removing As_*i*_. This cooking water percolation potentially maximizes the removal of As_*i*_, by both enhancing the volume of water the rice comes into contact with and by physically removing As_*i*_ leached from the rice. A number of variations on percolation technology are trialed; one where the water percolated through the rice is recycled through distillation; and one where the perfused water is discarded. The former is suitable where cooking water is itself elevated in As_*i*_ and/or where water is scarce or needs conserved, and the later suitable for low As_*i*_ water sources. The proposed technology can be low-tech and to demonstrate this in a domestic setting an off-the-shelf coffee percolator was used to remove As_*i*_ from wholemeal and polished rice.

## Materials and Methods

### Rice sourcing

Market rice was purchased from major UK retailers in the city of Belfast, or purchased online through UK retailers, *i*.*e*. all products tested were widely available to the UK populace. Of the 41 samples tested in this study 2 were generically labeled as being from the EU, 11 from Spain, 6 from Italy, 5 from Thailand, 5 from France, 2 from Egypt, 1 from Japan, 1 from Australia, 1 from Lebanon, 1 from Pakistan, 1 from Turkey and 5 from the USA; with 13 being unpolished (wholegrain) and the rest polished. Descriptions of samples are given in S1 Table.

### Cooking rice in different volumes of water

Initial experiments investigated the relationship between cooking water volume and As_*i*_ content. A standardized and repeatable approach was used where a 250 ml round bottomed Pyrex was used as the rice-cooking vessel. This was heated in an electrothermal heating mantle and the neck of the flask connected to a dimpled Vigreux 25cm condensing tube (14 rows of 3 dimples = 42 dimples in total) that returned steam as water to the flask. Evaporative losses as a confounding factor look in interpreting As_*i*_ content of cooked rice and by condensing any evaporated steam back into the cooking vessel such losses are negated. Rice (20g packet weight), from 6 wholemeal and 6 polished randomly selected from the bought packets, was placed in the flask and then double-distilled, deionized water added to give 3:1, 6:1 or 12:1 water:rice ratios. Rice was boiled until cooked and then removed from the heat. After cooling the sample was freeze-dried and then powdered using a Retch PM100 rotary ball-mill using a zirconium oxide lined vessel and zirconium oxide grinding balls. Uncooked rice was similarly freeze-dried and milled.

### Cooking rice in where percolating cooking water is recycled through condensation

Here all 41 samples of bought rice were cooked in a standard Soxhlet apparatus. Rice grain, fresh from the packet, was accurately weighed (2g) into a VWR Soxhlet thimble and was then placed into an 25cm long and 3.5cm diameter Quickfit Soxhlet which was attached to a 250ml receiving flask at one end and a dimpled Vigreux 25cm condensing tube (14 rows of 3 dimples = 42 dimples in total), with the receiving flask sitting within an electrically heated mantle and supported by a retort stand and clamps. At the start of the experiment the flask was filled with 200ml of double-distilled, deionized distilled water. Rice cooking was timed as per number of Soxhlet reflux cycles, with 3 cycles sufficient to fully cook the rice. At the end of the 3^rd^ cycle the thimble containing rice was removed and rice freeze dried and milled as described for the cooking volume experiment.

### Home-cooking in a coffee percolator

Six randomly chosen wholegrain and polished rice samples were cooked from the packet in a domestic environment, at quantities required to feed a large family, using an off-the-shelf coffee percolator with no adaptations. The coffee percolator chosen for these experiments was a catering model that is used to fill vacuum flasks, a Bravilor Bonamat. This type of percolator was used as it has a larger filter unit and it does not have a hotplate. Into the filter unit, lined with the manufacturers supplied paper filter, was placed 500g packet weight of the rice. The unit held 2L of water and this took 10 mins. to fully discharge through the filter unit. Twice times 10 mins. (*i*.*e*. 20 minutes of cooking) fully cooked polished rice, while 3 time 10 mins. (6L of water, *i*.*e*. 3* 2L) was needed for polished rice. Temperature of the water entering the filter was 98°C. The cooking experiments were conducted in triplicate for each rice type. The cooked rice was freeze dried and milled, as for other cooking experiments described above. Arsenic in the tap-water was low with only As_*i*_ being detected, at 0.050±0.02 μg/L, with the LOD being 0.036 μg/L.

### Chemical analysis

For As speciation powdered cooked and uncooked rice were weighed accurately to a weight of 0.1g into 50ml polypropylene centrifuge tubes to which 2ml of 1% conc. Aristar nitric acid was added and allowed to sit overnight. Batches of 40 samples were prepared which also include a black and rice CRM (NIST 1568b Rice flour) which has the arsenic species As_*i*_ and dimethylyarsonic acid (DMA) concentrations certified, then microwave digested in an CEM MARS 6 instrument for 30 min. at 95°C using a 3 stage slow heating program: to 55°C in 5 min. held for 10 min., to 75°C in 5 min., held for 10 min. to 95°C in 5 min., held for 30 minutes. The digestate, on cooling, was accurately diluted to 10ml with deionized distilled water. A 1 ml aliquot was transferred to a 2ml polypropylene vial and 10 ul of analytical grade hydrogen peroxide was added to convert any arsenite to arsenate to facilitate subsequent chromatographic detection. For multi-element analysis by ICP-MS, a more aggressive digestion procedure (heat to 95°C in 5 min. hold for 10 min. to 135°C in 5 min. then hold for 10 min., to 180°C in 5 min. then hold for 30 min.) was employed, with 2ml of concentrated Aristar nitric acid and 2ml hydrogen peroxide added and left to soak overnight before microwaving. Blanks and CRM NIST 1568b, which is certified for both arsenic speciation (As_*i*_ and DMA) and for a range of trace and macro elements, were included in each batch of 40 samples analysed.

To speciate As in rice the diluted 0.2% nitric acid digested rice solutions were run on a Thermo Scientific IC5000 Ion Chromatography (IC) system, with an Thermo AS7, 2x250mm column (and a Thermo AG7, 2x50mm guard column) and a gradient mobile phase (A: 20mM Ammonium Carbonate, B 200mM Ammonium Carbonate- Starting at 100% A, changing to 100% B, in a linear gradient over 15 min.) interfaced with a Thermo ICAP Q ICP-MS that monitored m/z^+^ 75, using He gas in collision cell mode. The resulting chromatogram was compared with that for authentic standards; DMA, As_*i*_, monomethylarsinic acid (MMA), tetratmethyl arsonium (TETRA) and arsenobetaine (AB). Arsenic present under each chromatographic peak was calibrated using a DMA concentration series.

Total elements were measured also using the Thermo ICAP Q but in direct solution acquisition mode. Rhodium was used as an internal standard. All elements reported were present both in calibration standards and in CRM NIST 1568b with only elements with good CRM recoveries reported. Additional elements were also analyzed by bench-top XRF (Rigaku CG) on powered samples. Only elements present in the CRM and with good analytical recoveries were presented.

### Statistics

General Linear Modeling (GLM), using Minitab v.16, was used to analyze the data. Residuals were tested for normality using the Anderson-Darling test, and where found to be normal so no transformation of the data was needed, except for percentage data, which were ranked and the ranks analysed.

## Results

The analysis of the As speciated CRM gave excellent recovery results, based on for n = 11, with 91.6±4.4% recovery for DMA and 88.2±3.3% recovery for As_*i*,_ the only two species found in that CRM that were also detectable in the rice analysed, giving a sum of species recovery of 90.3%. The CRM had a certified concentration of 0.182 and 0.092 mg/kg As for DMA and As_*i*_ respectively. The limits of detection (LOD) for both DMA and As_*i*_ (calculated from a DMA calibration) in rice was 0.0028 ±0.001 mg/kg DMA rice d.wt., n = 5. All samples presented were above LOD for As_*i*_ and only a few samples were below LOD for DMA (2), and in this case ½ LOD was used in statistical analysis of the data.

**Fig 1:**
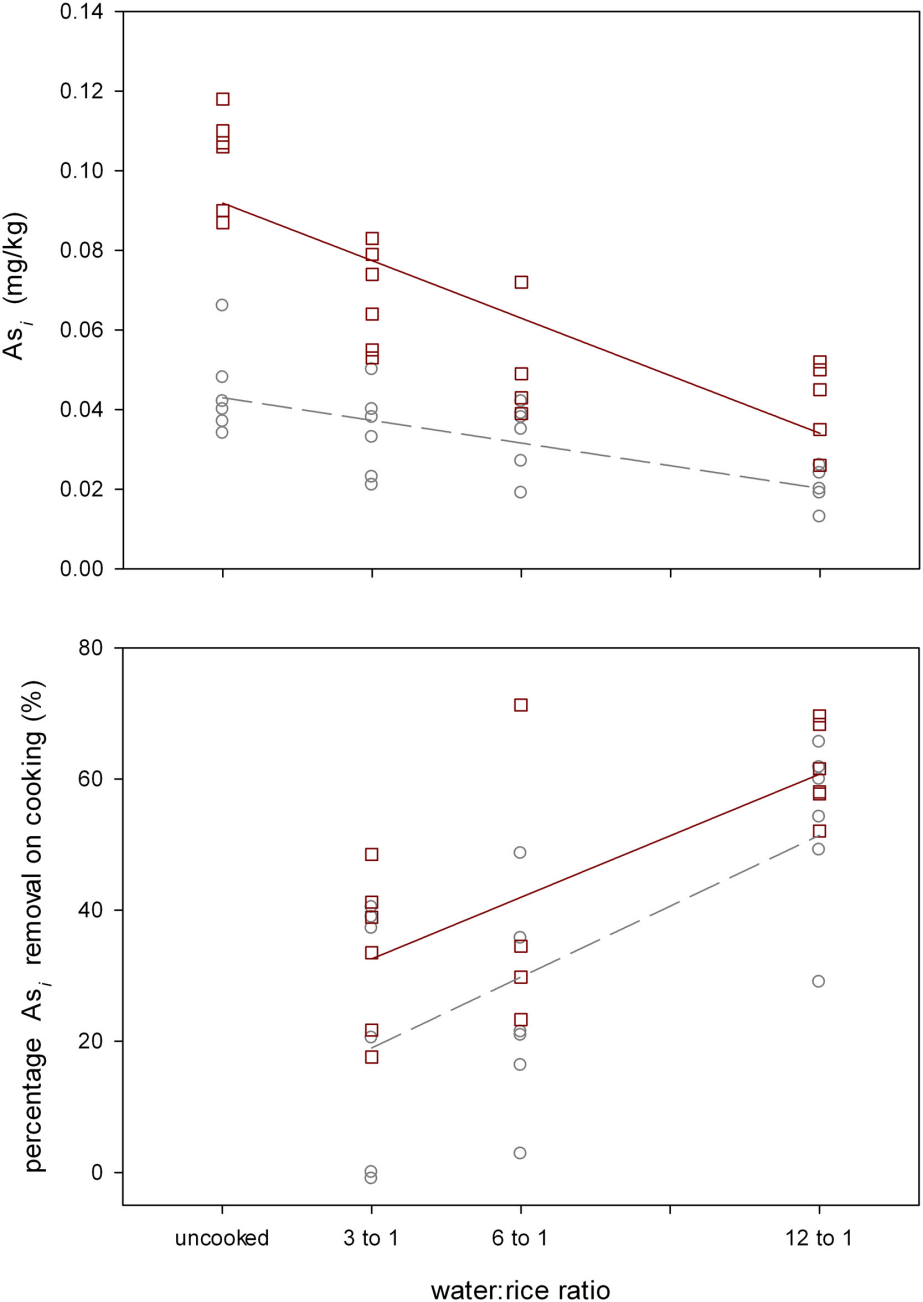
As_*i*_ concentration and percentage in rice that is either uncooked or has been cooked at different water: ratios. Squares are for wholegrain rice and circles for polished rice. Solid regression line is for wholegrain, dashed for polished.

The experiment that related As_*i*_ content of cooked rice to rice cooking volume found a linear relationship, where the greater the volume of cooking water the greater the removal of As_*i*_ from the cooked rice (Fig 1). GLM analysis found that whether the rice was wholegrain or polished (P<0.001), the volume of water used to cook the rice (P<0.001) and the interaction between rice type (wholegrain or polished) and cooking volume (P<0.001) were all highly significant with respect to the As_*i*_ content of the cooked rice. Uncooked wholegrain rice had higher As_*i*_ content in uncooked rice than polished, ~2-fold higher on average, but As_*i*_ was more efficiently removed from wholegrain, as observed in the steeper slope in the regression line and by the highly significant rice type*cooking volume interaction term. Up to 70% As_*i*_ could be removed at the highest water to rice ratio (12:1), with average removal of 57±5% across all rice types at the 12:1 ratio as compared to uncooked rice, and 53±5% and 61±3% for polished and wholegrain, respectively, with whole grain having higher initial inorganic arsenic concentrations, so more to remove. This figure for all rice was 29±5% and 29±4% at ratios of 3:1 and 6:1 respectively. DMA content was not reduced by cooking (S1 Fig), with only the rice type term being significant (P<0.001), reflecting the fact that wholegrain rice had higher concentrations of DMA than polished rice.

**Fig 2:**
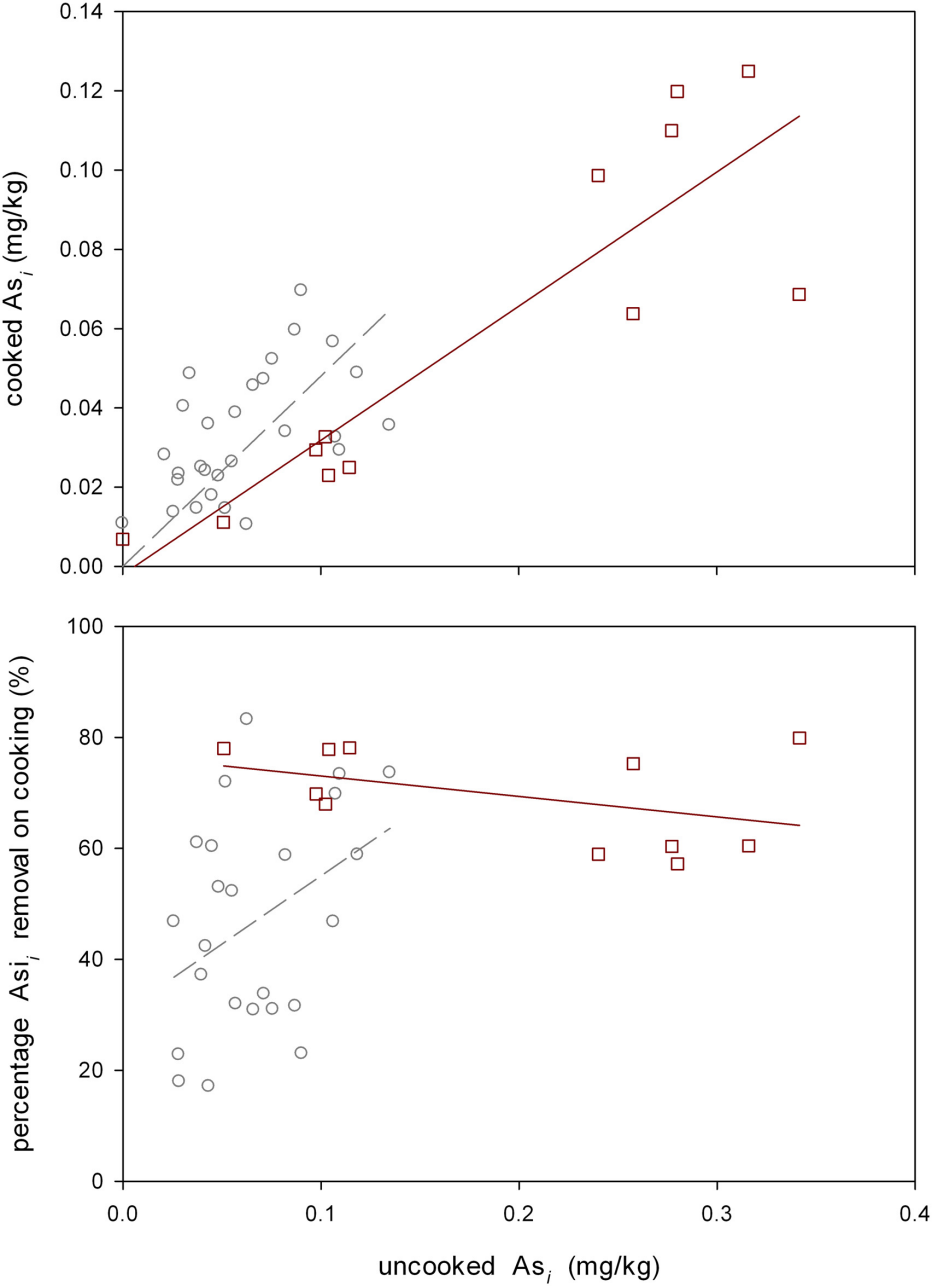
As_*i*_ concentration and percentage in rice cooked using a Soxhlet apparatus compared to uncooked rice. Squares are for wholegrain rice and circles for polished rice. Solid regression line is for wholegrain, dashed for polished.

For the Soxhlet cooking experiment it was found that this type of percolating cooking removed 59±8% and 69±10% of As_*i*_ compared to uncooked rice for polished and wholegrain, respectively (Fig 2). On the ranked percentage data (as percentages are not normally distributed) Soxhlet cooking As_*i*_ reduction was correlated with the concentration in the uncooked rice (P = 0.024), while the type of rice (wholegrain or polished) was highly significantly different (P = 0.001) and the rice type*As_*i*_ content of uncooked rice interaction term was significant in the analysis of co-variance GLM analysis, with As_*i*_ in uncooked rice being the covariate. For the absolute As_*i*_ concentrations in cooked rice, the As_*i*_ in uncooked rice covariate term was highly significant (P<0.001) while the rice type and the interaction term were not significant. In some individual samples up to, and in one case over, 80% of As_*i*_ could be removed by percolation, for both wholegrain and polished rice. For polished rice there is a trend that the more As_*i*_ present in the uncooked rice the more As_*i*_ is removed by cooking. This was not the case for wholegrain, where there was a very slight trend in the opposite direction, with the difference between polished and wholegrain probably due to the whole grain rice having higher inorganic arsenic in the raw rice, and that inorganic arsenic removal saturates at the higher initial raw rice concentrations. For DMA (S2 Fig) only the DMA content of uncooked rice was significant in the GLM analysis of covariance (P<0.001), and no terms were significant for the percentage of DMA removal. This illustrates that DMA is very poorly removed on rice cooking, even under the most stringent conditions of this Soxhlet based cooking method.

**Fig 3:**
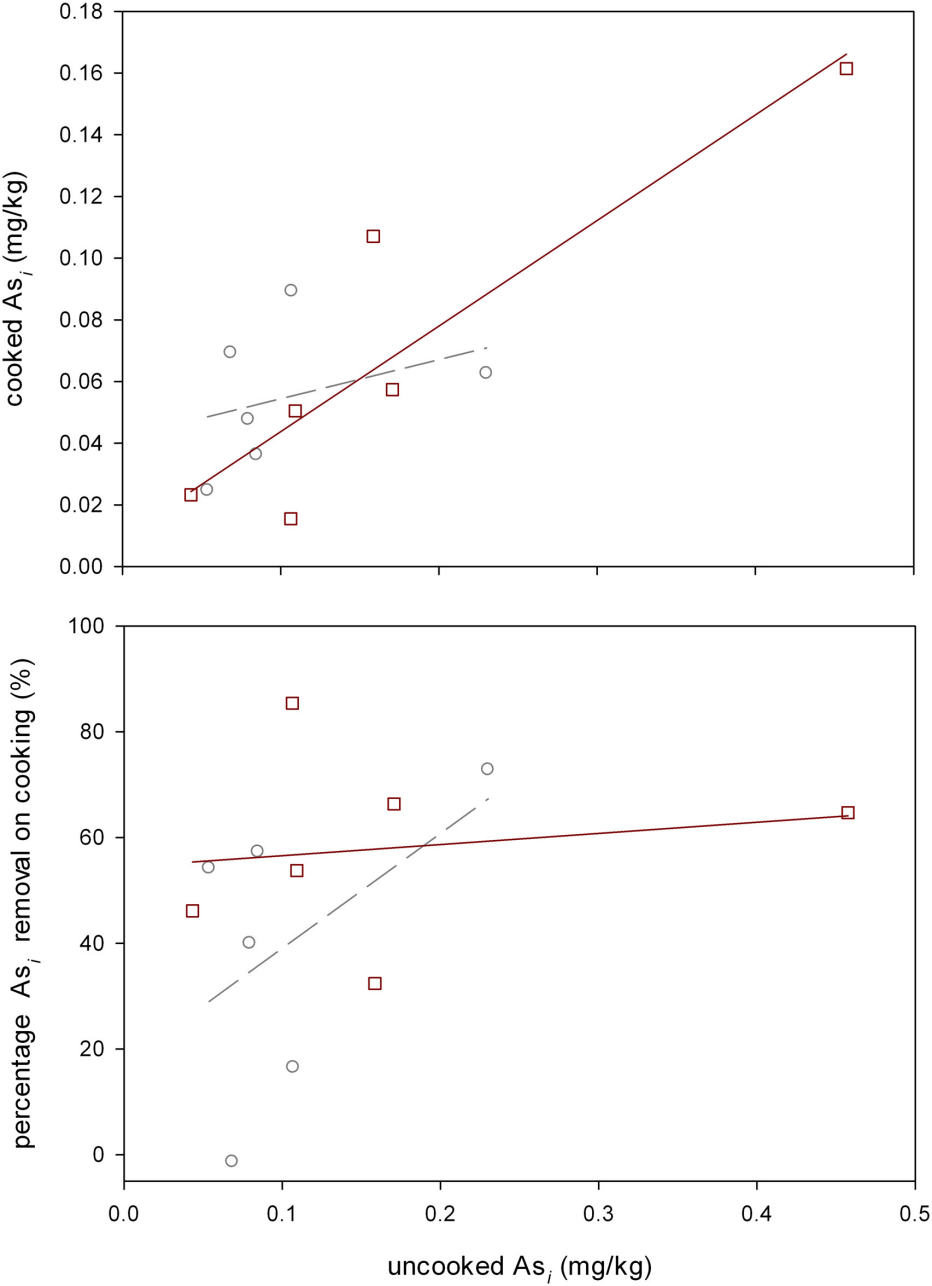
As_*i*_ concentration and percentage in rice cooked using a coffee percolator compared to uncooked rice. Each point is the mean of 3 replicates. Squares are for wholegrain rice and circles for polished rice. Solid regression line is for wholegrain, dashed for polished.

**Fig 4:**
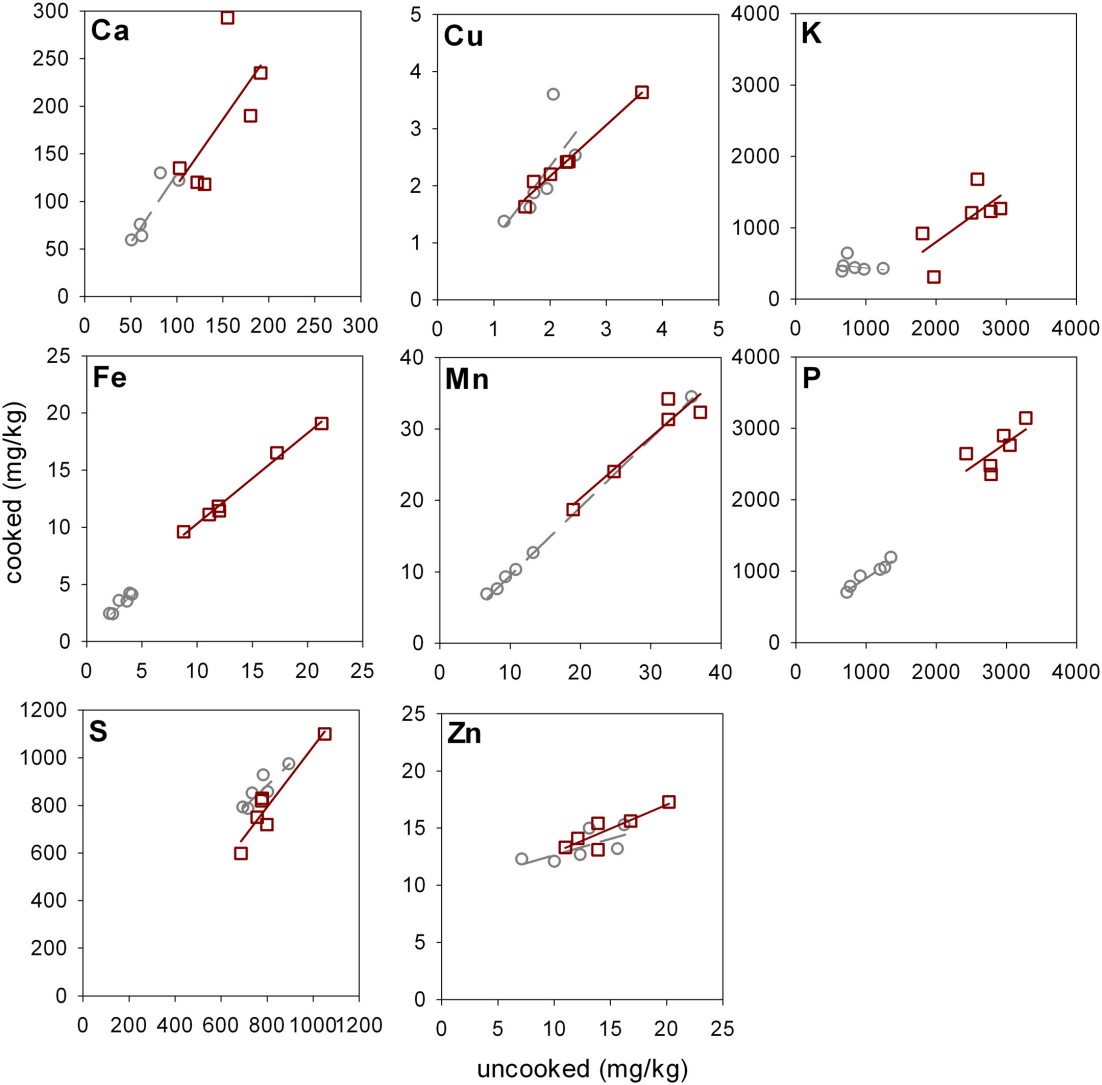
Total calcium, copper, iron, manganese, phosphorus, silicon, sulphur and phosphorus concentrations in rice cooked using a coffee percolator compared to uncooked rice. Each point is the mean of 3 replicates. Squares are for wholegrain rice and circles for polished rice. Solid regression line is for wholegrain, dashed for polished.

When a home cooking with a coffee percolator was trialed 49±7% of As_*i*_ could be removed across all 12 samples, with up to 85% removal observed for one sample (Fig 3). GLM analysis of covariance showed that only the initial As_*i*_ concentration in the uncooked rice had a significant contribution to the actual concentration in the cooked rice (P = 0.045). This figure for DMA (S3 Fig) was (P<0.001). Terms containing rice type were non-significant for both As_*i*_ and DMA. None of the terms were significant for the percentage As_*i*_ removal experiments, showing that rice type was not a contributing factor in As_*i*_ removal in these coffee percolating cooking experiments. A pair t-test showed that As_*i*_ concentration was significantly (P = 0.009) reduced on cooking. There was no significant reduction in DMA content between cooked and uncooked rice (P = 0.263). When a range of trace and macro-elements were analyzed between coffee percolator cooked and uncooked only potassium (P<0.001) and phosphorus (P = 0.016) were significantly different, while calcium, copper, iron, manganese, sulphur and zinc were non-significant (Fig 4). For potassium 53% was lost on cooking. For phosphorus this figure was 7%.

## Discussion

Mitigating As_*i*_ levels in rice is essential as this commodity is the major global source of this carcinogen, with As_*i*_ derived from rice consumption predicted to cause significant increases in excess lifetime cancer burdens, in the order of ~1–2 in 1,000, for the highest rice eating communities [1]. Using a continual flow of clean cooking water *versus* traditional static systems enables up to 85% of the As_*i*_ content of raw grain to be removed on cooking, much higher than the use of standard static cooking water or steaming based systems [10]. Furthermore, this continual flow system offers a way of assaying which rice is amenable to have As_*i*_ removed on cooking, and using a Soxhlet based system, the method is also standardized and repeatable in any laboratory and is not reliant on the purity of water used because delivery of cooking water is through distilled steam. Although the standardized cooking methods were developed around reflux/Soxhlet for standardization, household or commercial scale cookers could adopt the same approach as demonstrated here in the coffee percolator experiment.

The development of commercial approaches based on continuously flushing rice with arsenic free cooking water would have application in baby rice manufacture, for example, as baby rice is of particular concern with respect to human exposures [12,13] and baby rice is a product where grain is pre-cooked, dried and milled, much the same as the sample preparation for analysis here. Rice milk is similarly problematic, being elevated in As_*i*_, and is fed to lactose intolerant children in quantities that raise concern [14]. Rice milk is prepared from cooked rice and manufacturing processes could be optimized for As_*i*_ cooking using the continual cooking water flow principle demonstrated here. It is not difficult to envisage that large-scale rice cookers for baby rice and rice milk manufacture could be based either on the distillation approach, as demonstrated here by using a Soxhlet system, or with a continual flow of externally pre-heated water passing through the rice, as demonstrated here by the commercial coffee percolator approach. With baby rice if elemental nutrients (*i*.*e*. potassium) do need supplementation this is easy to do and, indeed, many baby rice products are nutrient and vitamin fortified. Vitamin removal was not assessed here and again may need to be investigated with respect to commercial production of products such as baby rice, and as rice is an important source of soluble B-vitamins [15], with vitamin B2 known to be leached from rice on cooking dependent on processing methodologies [16].

Non-milled, pre-cooked rice is becoming an important market in the prepared food business, from stand-alone precooked boiled rice to boiled rice in pre-packed Asian & S.E. Asian foods, and in products such as pre-cooked baby food jars. For all these products continual cooking water flow cooking approaches could be applied to lower As_*i*_ to consumers. Cooked rice texture using continual flow cooking, not assessed here, while unimportant for milled baby rice, would be important for non-milled pre-cooked products, but manufactures could optimize rice type/source and cooking conditions to optimize final texture of their product. Home cooking continual flow would need the specific product design, but should be achievable, and given that there is already a wide penetrance of specific rice cookers for domestic use, based on steaming, so there is a market for specific rice cookers. We have demonstrated here that coffee percolator technology could also be used to cook rice, and that such technologies could be optimized for As_*i*_ removal and, and to optimize the heat and water usage to most efficiently achieve this goal. The coffee percolator technology is most suitable for cooking rice in regions that have low As_*i*_ available for the cooking water, although distilled/purified water could be directly used if in high water As_*i*_ regions, if available. Where As_*i*_ is high in cooking water then the recycling of water through continuous distillation is the solution. The key to a home based continual reflux distillation rice cooker development simply needs a steam condensers unit to supply water to rice suspended in a basket with a bottom receiver (pot) directly heating the water. Condensers could be based on water/ice cooling, on thermal properties of materials used for a lid with the design of the inside of the lid optimized to deliver stream of condensing water to the rice, or on electro-thermal cooling technologies for the lid/condenser. Home-based appliances for water/ethanol extraction of essential oils from plant-based material are already available [17] and could be readily adapted, to lower cost and to increase efficiency.

*In situ* speciation [6] shows that grain As_*i*_ is either as free As_*i*_ or as sulfhydryl complexed As. It is not clear why grains with more As_*i*_ release more As_*i*_ on cooking as compared to those low in As_*i*_, but perhaps it is grain geometry, *i*.*e*. that As_*i*_ in the outer portions of the grain are more easily removed than those in the inner portions. It is known that As_*i*_ is more elevated at the grain surface than in the centre [6].

The finding that As_*i*_ is readily leached from rice while DMA is not conforms well with what is known with their rice gut availability where As_*i*_ has a high gut availability where DMA does not [18]. The fact that As_*i*_ is readily leached from both wholegrain and polished rice is also important. Although not tested here, if rice bran As_*i*_ is readily removed using continual flow of replenished cooking water then this product which is highly elevated in As_*i*_ may be made suitable for the human food-chain, given that in many aspects it is a highly nutritious product whose health and commercial value is negated by its As_*i*_ content.

## Supporting Information

Three additional graphs, pertaining to the DMA content of rice that complement the As_*i*_ content of rice shown in S1–S3 Figs in the main body of the text. A table of rice sample descriptions is also given in S1 Table.

S1 Fig DMA concentration and percentage in rice that is either uncooked or has been cooked at different water: ratios. Squares are for wholegrain rice and circles for polished rice. Solid regression line is for wholegrain, dashed for polished.
(TIF)

S2 Fig DMA concentration and percentage in rice cooked using a Soxhlet apparatus compared to uncooked rice. Squares are for wholegrain rice and circles for polished rice. Solid regression line is for wholegrain, dashed for polished.
(TIF)

S3 Fig DMA concentration and percentage in rice cooked using a coffee percolator compared to uncooked rice. Each point is the mean of 3 replicates. Squares are for wholegrain rice and circles for polished rice. Solid regression line is for wholegrain, dashed for polished.
(TIF)

S1 Table Rice sample descriptions. (DOCX)
