# Supplementary material for: Rethinking Rice Preparation for Highly Efficient Removal of Inorganic Arsenic Using Percolating Cooking Water
Source: PLoS One. 2015 Jul 22;10(7):e0131608. doi: 10.1371/journal.pone.0131608 (PMC4511802; doi:10.1371/journal.pone.0131608)
Supplement: S1 Table — (DOCX) [file pone.0131608.s004.docx]

**S1 Table** Rice sample descriptions.
